# The Impact of COVID-19 Lockdown on Patients with Obesity after Intensive Cognitive Behavioral Therapy—A Case-Control Study

**DOI:** 10.3390/nu13062021

**Published:** 2021-06-11

**Authors:** Simona Calugi, Beatrice Andreoli, Laura Dametti, Anna Dalle Grave, Nicole Morandini, Riccardo Dalle Grave

**Affiliations:** Department of Eating and Weight Disorders, Villa Garda Hospital, Via Monte Baldo, 89, 37016 Garda, VR, Italy; si.calugi@gmail.com (S.C.); drandreolibeatrice@gmail.com (B.A.); lauradametti96@gmail.com (L.D.); annadallegrave@gmail.com (A.D.G.); nicolemorandini94@gmail.com (N.M.)

**Keywords:** lockdown, cognitive behavioral therapy, treatment outcomes, COVID-19, isolation

## Abstract

Background: The COVID-19 lockdown may have negatively impacted the treatment of obesity. This study aimed to assess the effect of COVID-19 lockdown in patients with obesity treated with intensive residential cognitive behavioral therapy (CBT-OB). Methods: This retrospective case-control study analyzed 129 patients with severe obesity who experienced COVID-19 lockdown in the 6 months after discharge from intensive residential CBT-OB, comparing their outcomes on weight loss, binge-eating episodes, and general health status with those in a sample of patients matched by gender, age, and body mass index given the same treatment before the COVID-19 outbreak as control. Patients were assessed at baseline and by phone interview 6-month follow-up. Results: Both groups had lost more than 9% of their baseline bodyweight and reported a significant decrease in binge-eating episodes and similar general health status at 6-month follow-up. However, control patients achieved a significantly greater weight loss than those who experienced lockdown, although half of lockdown patients reported persisting with CBT-OB procedures after their discharge. Conclusion: Patients with obesity treated with CBT-OB and exposed to COVID-19 lockdown, despite achieving lower weight loss than non-exposed patients, had a healthy weight loss at 6-month follow-up and comparable reduction in binge-eating behaviors.

## 1. Introduction

As a result of the coronavirus (COVID-19) pandemic, Italy was in lockdown from 9 March to 11 May 2020. During the lockdown, any medical activity considered non-urgent was suspended, including treatment for obesity. This inevitably caused a high risk of relapse in people with obesity, potentially aggravated by negative emotions associated with concerns related to the severe complications of COVID-19 infection reported in this fragile population. Indeed, several recent published commentaries have highlighted the association between obesity and increased COVID-19 disease severity [1,2,3,4]. This association has been confirmed by a meta-analysis, which identified nine studies, all confirming that patients with obesity had more severe presentation of COVID-19 and worse outcomes [5].

Some papers have also suggested that social isolation could have a negative effect on the management of obesity. In particular, an editorial has pointed out the negative role of forced lockdown in these patients, since social isolation is a profound characteristic feature of people with severe obesity, as well as a factor aggravating the stigma of obesity [6]. Similarly, other papers have commented on the potential role of the COVID-19 pandemic in worsening the obesity “epidemic”, in particular due to the behavioral changes imposed by the social restrictions necessary to contain the spread of the virus. Indeed, home confinement and potential associated mental problems such as anxiety and depression could prompt increases in food intake, especially if people with obesity are exposed to a large amount of food stored at home during lockdown, as well as forced physical inactivity [7,8,9,10,11] (during the period in question, it was illegal in Italy to leave the house for exercise, except in order to walk one’s dog).

Some studies have empirically investigated the impact of lockdown and the role of the COVID-19 pandemic on lifestyle changes [12], psychosocial life [13], anxiety and depression [14], and weight change [15] in patients with obesity. In general, it has been found that the COVID-19 pandemic and its consequences in terms of social isolation had a significant negative impact on patients with obesity and their treatment, regardless of their infection status. However, the main limitation of these studies was that they used retrospective self-report questionnaires, telephone interviews, or chart reviews and included only patients with obesity, without a comparison with patients with obesity who had not been exposed to lockdown; such study designs are not able to evaluate the real role of isolation on weight management or quality of life in patients with obesity exposed to lockdown.

Since the COVID-19 emergency is not yet over, more data on the effect of lockdown on the management of patients with obesity are needed in order to design specific strategies and procedures to support them during such difficult periods. Hence, our study was designed to evaluate the potential impact of lockdown on weight management, eating behaviors, and lifestyle in patients with severe obesity treated with intensive residential cognitive behavior therapy for obesity (CBT-OB) [16] and reassessed after 6 months of follow-up encompassing the COVID-19 lockdown period, as compared to those who completed the 6-month follow-up before the start of the lockdown. Our research hypothesis is that patients with severe obesity treated with intensive residential CBT-OB and exposed to COVID-19 lockdown achieve lower weight loss than non-exposed patients but, despite this, can maintain a successful weight management.

## 2. Materials and Methods

### 2.1. Intensive Residential CBT-OB

Intensive residential CBT-OB is a specialized treatment for severe obesity lasting 21 days. The residential treatment is based on the personalized CBT-OB described in previous publications [16,17] and includes three main areas of intervention: (i) a low-calorie diet (25% protein, 20% fat, and 45% carbohydrates) designed to produce a 500 kcal energy deficit per day, and daily multivitamin supplements; (ii) a physical activity program including 30 min per day of indoor cycling, and two 45 min sessions per week of calisthenics; and (iii) a CBT intervention consisting of 15 group sessions addressing the following topics: (a) self-monitoring of food intake, physical activity, and bodyweight; (b) stimulus control strategies (in particular how to reduce food stimuli at home); (c) proactive problem solving to address events influencing eating and associated mood changes; (d) cognitive restructuring of dysfunctional thoughts that hinder weight loss, to develop a stable weight control mindset; and (e) relapse prevention skills. The patients received a written manual describing the strategies and procedures involved [18], and their significant others participated in two group sessions designed to impart information about obesity and how to create an optimum home environment to facilitate weight management.

**Figure 1:**
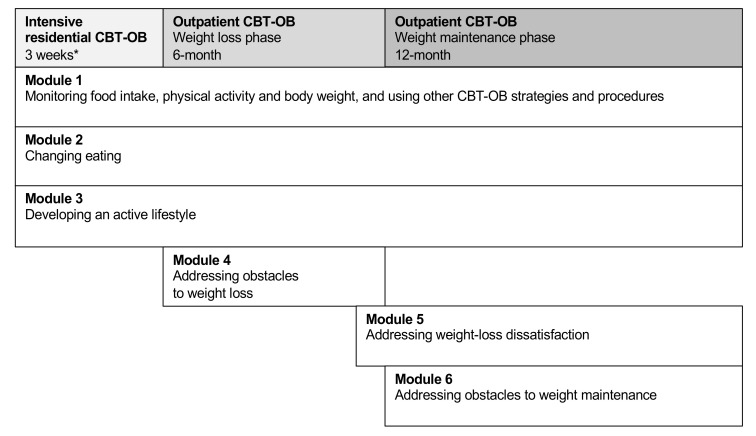
The map of cognitive behavioral therapy for obesity (CBT-OB). * Patients treated in residential CBT-OB start the first three modules of CBT-OB in this intensive setting, and then they are offered to continue the treatment with outpatient CBT-OB.

As is routine in our unit, after the intensive residential CBT-OB program, patients were offered the possibility of participating in 18 months of outpatient treatment, with the first 6 months focused on weight loss and the following 12 months on weight maintenance (Figure 1).

### 2.2. Study Design and Participants

**Figure 2:**
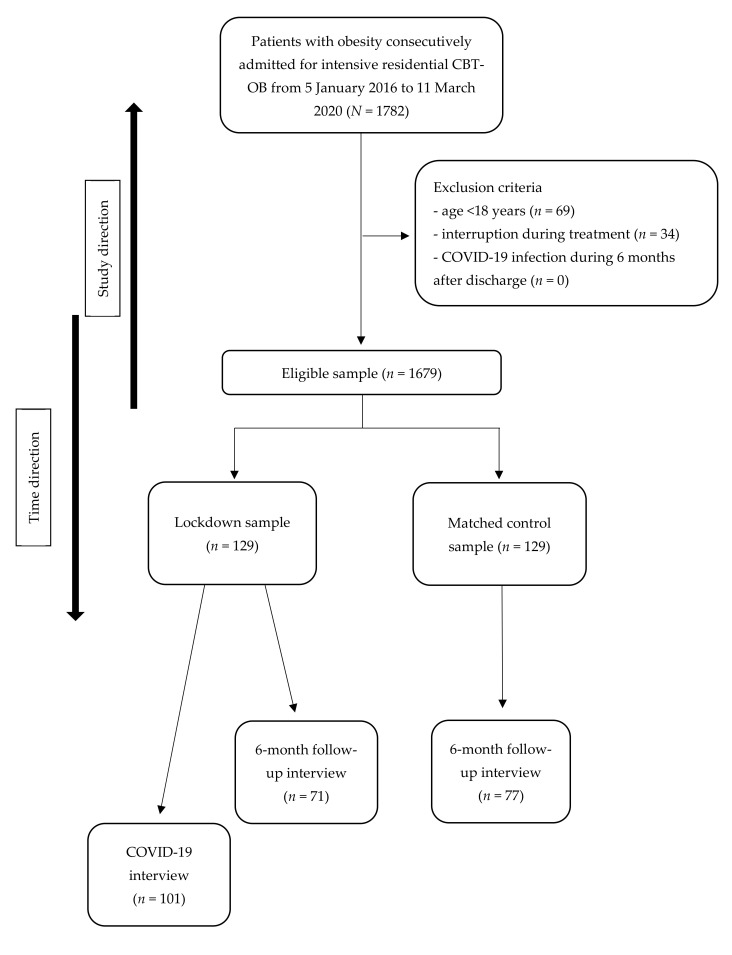
Flowchart.

This was a case-control retrospective study with a gender-, age-, and body mass index (BMI)-matched control group. The global sample included all patients consecutively admitted to the Villa Garda Hospital Department of Eating and Weight Disorders (Figure 2) who met the following criteria: (i) BMI ≥ 30 kg/m^2^; and (ii) residential treatment, indicated by a global score >25 on the Comprehensive Appropriateness Scale for the Care of Obesity in Rehabilitation (CASCO-R) scale [19]. The inclusion criteria to be eligible for the study were (i) age ≥ 18 years; (ii) having completed the intensive residential CBT-OB at Villa Garda Hospital; and (iii) producing a signed informed consent. Patients were excluded from the study if they had medical comorbidities and/or were taking drugs influencing bodyweight, and if they had COVID-19 during treatment or in the following 6 months.

The sample exposed to COVID-19 lockdown was recruited from patients admitted to the Villa Garda Hospital between 6 November 2019 and 11 March 2020. This decision was based on the consideration that all or part of the 6 months following discharge from intensive residential CBT-OB (up to the 6-month follow-up interview) spanned the lockdown, as a consequence of the extraordinary measures adopted by the Italian government to contain the COVID-19 pandemic.

The control sample, matched by gender, age (±2 years), and BMI (±2 kg/m^2^), was recruited from patients admitted to Villa Garda Hospital from January 2016 to August 2019, a period during which the patients did not suffer the effects of lockdown in the 6 months following discharge from intensive residential CBT-OB.

Patients were informed at the time of the COVID-19 interview about the purposes of the study and the nature of the analysis performed. No patients received any remuneration for their participation, and all signed informed consent to participate in the study. The research protocol was approved by the Verona and Rovigo Ethics Committee (Project identification code 57007).

### 2.3. Assessment

Case report form, measured bodyweight and height, appropriateness of residential treatment, eating disorder psychopathology and behaviors, and general psychiatric features were collected on the first day of admission to the intensive residential CBT-OB (baseline). Bodyweight and eating disorder psychopathology and behaviors were also assessed at the end of the intensive residential CBT-E. A follow-up interview was conducted and self-reported bodyweight was collected at 6 months after discharge. Finally, lockdown patients were interviewed by phone between October and November 2020 with ad hoc COVID-19 interviews (Appendix A in the back matter).

#### 2.3.1. Case Report Form

This was used to record demographic and weight data and weight and diet history. It was filled out by the physicians who directly interviewed the patients on the first day of admission to the intensive residential CBT-OB (baseline).

#### 2.3.2. Bodyweight and Height

Bodyweight was measured on a calibrated scale (Seca digital wheelchair scale Model 664, Hamburg, Germany) at baseline, with patients wearing no shoes and only lightweight clothes. Height was measured at baseline using a stadiometer (Wunder wall-mounted mechanical height rod Model 00051A, Milan, Italy). BMI was calculated via the standard formula of bodyweight in kilograms divided by height in meters squared. Bodyweight and height were measured at admission and discharge and were self-reported at 6-month follow-up.

#### 2.3.3. Appropriateness of Residential Treatment

This was assessed using the CASCO-R scale, which was jointly developed by the Italian Society of Obesity (SIO) and the Italian Society for the Study of Eating Disorders (SISDCA) to assess the suitability of different settings of care in Italy (i.e., residential rehabilitation, intensive outpatient rehabilitation, or outpatient treatment). The scale comprises four sections: (i) BMI and waist circumference; (ii) comorbidity associated with obesity; (iii) risk factors potentially increasing obesity-related morbidity; and (iv) previous hospitalization for metabolic/nutritional rehabilitation. Each item is assigned a score (with negative scores for one or more hospital stays in nutritional rehabilitation units), and a global score of >25 indicates a condition of severe obesity that would benefit from residential treatment. The global CASCO-R score is significantly correlated with both overall workload and adverse clinical event measures and has excellent internal validity and test–retest reliability [19].

#### 2.3.4. Eating Disorder Psychopathology and Behaviors

Patients were assessed at baseline and at discharge from intensive residential CBT-OB using the validated Italian version of the Eating Disorder Examination Questionnaire (EDE-Q) [20,21]. The EDE-Q is a self-report questionnaire for assessing eating disorder psychopathology and behaviors in the 28 days before interview. It scores the behavioral symptoms of individuals with eating disorders, including binge eating, self-induced vomiting, laxative misuse, and excessive exercising. Moreover, EDE-Q includes four subscales (Restraint, Eating Concern, Weight Concern, and Shape Concern) and a global score for eating disorder psychopathology. Items are rated on a 7-point forced-choice scale (range 0–6). The Italian version of the EDE-Q has demonstrated excellent criterion validity and high test–retest reliability (r = 0.80), and its global score has very good inter-rater reliability (rho = 0.97) [22]. In our sample, the Cronbach’s alpha of the EDE-Q global score was 0.83.

#### 2.3.5. General Psychiatric Features

The Global Severity Index (GSI) of the validated Italian version of the Symptom Check List-90-Revised (SCL-90-R) [23,24] was used to assess patients’ general psychiatric features at baseline. The SCL-90-R comprises 90 items, each of which is scored on a 5-point scale of distress (0–4), ranging from “not at all” to “extremely”. A global score was calculated and used in this study.

#### 2.3.6. 6-Month Follow-Up Interview

All patients were contacted to receive a telephone interview 6 months after discharge by researchers not involved in the treatment delivery. The interview, which is part of the standard assessment protocol in our unit, investigates the current bodyweight, the lowest weight reached after the last contact with our unit, and satisfaction with weight loss. It also records whether the patient followed a post-discharge treatment for weight loss and investigates their general health status, mood, and any binge-eating episodes in the previous 28 days. Binge-eating episodes were scored as follows: 1 = less than 1 episode per week; 2 = 1 episode per week; 3 = 2–3 episodes per week; 4 = 4–7 episodes per week; 5 = 8–13 episodes per week; 6 = 14 or more episodes per week.

#### 2.3.7. COVID-19 Interview

The lockdown patients were interviewed by phone between October and November 2020 by researchers not involved in the treatment delivery. Our ad hoc COVID-19 interview (Appendix A) was designed to retrospectively investigate work conditions, food and eating management (being worried about not having enough food available and the accumulation of food), and the application of CBT-OB procedures for weight management (the regular eating procedure (i.e., eating three planned meals and two snacks and not eating between), adhering to calorie goals, maintaining an active lifestyle) during COVID-19 lockdown.

### 2.4. Statistical Analysis

Descriptive statistics are reported as means and SD for continuous variables and as percentages for categorical variables. The *t* test for independent samples or Mann–Whitney U test for continuous variables, as appropriate after test for normality, and the chi-squared test for dichotomous variables were used to compare lockdown and control groups in terms of baseline clinical and demographic variables and clinical variables measured at follow-up.

The change in bodyweight in both groups from baseline to 6-month follow-up was investigated using repeated-measures analysis of variance (RMANOVA). Pairwise comparisons with post hoc Bonferroni were carried out. Moreover, we tested the homogeneity of variance assumption using Mauchly’s sphericity test. The RMANOVA is presented for both completers and intention to treat. For intention-to-treat analysis, we handled missing bodyweight data at follow-up using a multiple imputation procedure with the fully conditional specification method.

All statistical analyses were carried out using SPSS software (IBM SPSS Statistics for Windows, Version 27.0. Armonk, NY, USA)

## 3. Results

Demographic and clinical features of both lockdown and control patients are presented in Table 1. Both groups were 70% women, and both had a mean age of about 57 years and a BMI around 42 kg/m^2^. Comparison between the two groups indicated that they had similar mean age and BMI, and identical gender distribution, and that there were no significant differences in eating disorder or general psychopathology or eating=disorder behavior scores.

### 3.1. Follow-Up Completion

All selected patients who completed the three weeks of intensive residential CBT-OB were contacted after 6 months to participate in the follow-up interview. A total of 71 lockdown patients (55.0%) and 77 control patients (59.7%) agreed to respond to the telephone follow-up interview; 2 (1.6%) and 4 (3.1%), respectively, refused telephone contact; and 56 (43.3%) and 48 (37.2%), respectively, were not found or furnished unreliable data (chi-squared = 1.25, *p* = 0.466). No significant differences in demographic or baseline clinical variables were found between patients who participated in the follow-up interview and those who did not. The only exception was that among control patients, respondents had a significantly higher age than non-respondents to follow-up interview (respondents 59.6 (SD = 10.8) years vs. non-respondents 51.9 (SD = 16.9) years, *p* = 0.005).

### 3.2. Response to Treatment

**Figure 3:**
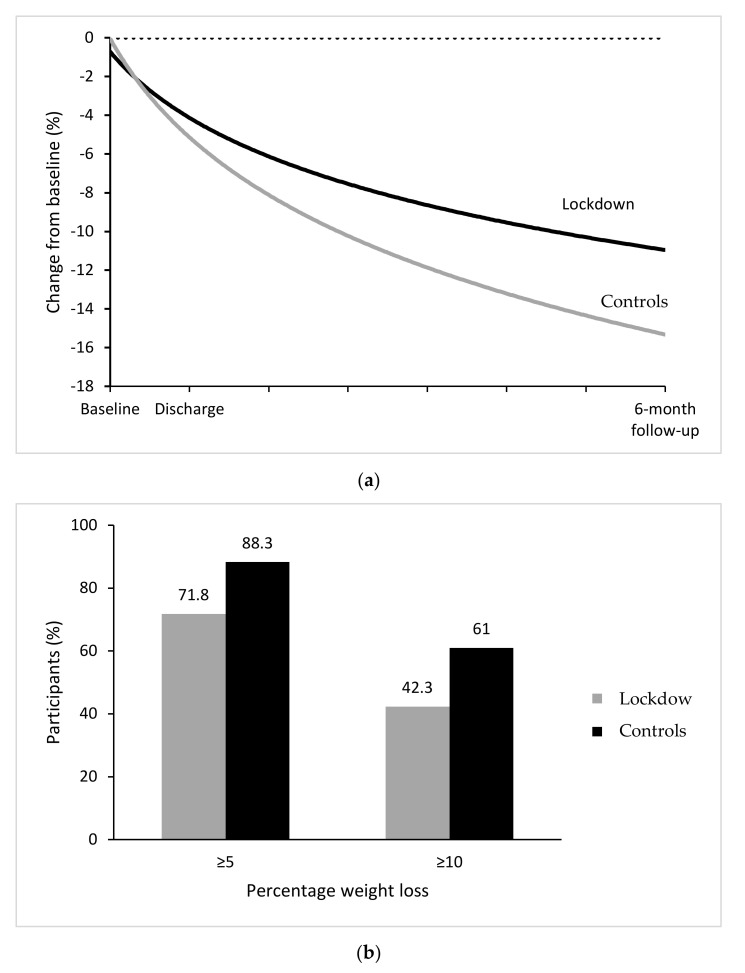
(**a**) Observed mean percentage change from baseline in bodyweight over time among patients with severe obesity exposed and not exposed to COVID-19 lockdown. (**b**) Observed percentages of patients exposed and not to COVID-19 lockdown who had bodyweight reductions of at least 5% and 10% from baseline to 6-month follow-up. Percentages are based on the number of participants for whom data were available at 6-month follow-up: 71 exposed patients (lockdown) and 77 not exposed (control) patients.

Table 2 shows bodyweight and BMI at each time point, and weight change from baseline to discharge and 6-month follow-up in the two groups, using both completer and intention-to-treat analysis. In completers, ANOVA for repeated measures indicated a significant change over time and a significant TimeXGroup interaction in both bodyweight and BMI, indicating a significant weight loss in both samples, but a greater mean weight loss in control than in lockdown patients at 6-month follow-up. Pairwise comparisons with post hoc Bonferroni indicated that overall bodyweight and BMI were significantly higher at admission than at discharge and significantly higher at discharge than at 6-month follow-up. While from baseline to discharge, the two groups achieved similar weight loss (a percentage of around 4%), from baseline to 6-month follow-up, control patients reported a greater percentage of weight lost (13.0%) than those who had experienced lockdown (9.4%, *p* = 0.004), indicating that the larger bodyweight change difference occurred from discharge to 6-month follow-up (Figure 3a). In fact, at discharge, similar proportions of lockdown and control patients displayed 5% and 10% weight loss, but these proportions were significantly different at 6-month follow-up, with a higher proportion of control patients reporting 5% and 10% weight loss than lockdown patients (88.3% vs. 71.8% and 61.0% vs. 42.3%, respectively) (Figure 3b). Similar findings were found for intention-to-treat analysis.

To evaluate the change in binge-eating episodes from baseline to follow-up, the binge-eating episodes assessed at baseline with the EDE-Q were characterized in the same way as the binge-eating episodes assessed at the follow-up interview. Table 3 shows binge-eating episodes at baseline and 6-month follow-up in lockdown and control patients and indicates that there was no difference between groups in the proportion of binge-eating episodes, either at admission or at 6-month follow-up.

Finally, considering the questions asked to the patients at the 6-month follow-up interview, lockdown and control patients reported similar rates of post-inpatient treatment (79.7% vs. 78.7%, respectively, chi-squared = 0.02, *p* = 0.878), but a significantly higher percentage of lockdown than control patients interrupted it (44.3% vs. 15.0%, respectively, chi-squared = 12.39, *p* < 0.001). Both sets of patients reported a similar general health status (chi-squared = 7.99, *p* = 0.092), but a significantly greater proportion of control than lockdown patients indicated that they had almost always or always felt happy or joyful in the past four weeks (chi-squared = 15.13, *p* = 0.010).

### 3.3. COVID-19 Interview for Lockdown Patients

Out of the 129 adult patients admitted to Villa Garda Hospital between 6 November 2019 and 11 March 2020 who completed the intensive residential CBT-OB, 101 (78.3%) agreed to participate in the COVID-19 interview, 7 (5.4%) refused to participate, and 21 (16.3%) were not available for comment. All 101 patients answered all questions.

Regarding their job situation, among the 56 patients who were working before the COVID-19 lockdown, 35 patients (62.5%) did not stop working or lose their job, while 19 (30.4%) lost their job or temporarily stopped working. Among those who continued working, 17 (48.6%) continued to work outside the home, 17 (48.6%) remotely from home (smart working), and 1 did both.

Concerning food and eating management, 7 out of 101 (6.9%) respondents declared that during the COVID-19 lockdown, they were worried about not having enough food available, and 34 (33.7%) accumulated more food in the house than normal. As regards the application of CBT-OB procedures for weight management, 30 (29.7%) lockdown patients declared that they never or rarely adhered to the regular eating procedure, 21 (20.8%) sometimes, and 50 (49.5%) often or always. Moreover, 35 (34.7%) never or rarely stuck to their recommended daily calorie goals, 17 (16.8%) sometimes, and 49 (48.5%) often or always. Finally, 39 (38.6%) respondents reported that they never or rarely exercised, 13 (12.9%) sometimes did, and 49 (48.5%) often or always did. See details in Table 4.

## 4. Discussion

This study evaluating the potential impact of COVID-19 lockdown on weight loss, binge-eating episodes, general health status, and mood status in patients with severe obesity treated with intensive residential CBT-OB and re-assessed 6 months after discharge had four main findings. The first is that patients whose post-discharge period coincided with the national lockdown achieved a significantly lower weight loss at 6-month follow-up than those who completed the 6-month follow-up before the COVID-19 pandemic. This suggests that the COVID-19 lockdown may have played a role in hindering weight loss in patients with obesity and confirms data from previous studies conducted without a control group for comparison [15,25,26,27,28,29,30]. The negative impact of lockdown in patients with obesity also seems to be confirmed by our data showing that a significantly higher percentage of lockdown versus control patients interrupted outpatient treatment after intensive residential CBT-OB, even though a similar percentage of patients in both groups (about 80%) declared having started it. The second finding is that, despite the above difference, both groups achieved a substantial and healthy reduction in bodyweight. Specifically, the mean weight loss among completers after 6 months was 9.4% and 13.0% in lockdown and control patients (9.9% and 12.3% on intention-to-treat analysis), respectively. That being said, among completers, a greater percentage of control patients reported 5% and 10% weight loss at 6 months than lockdown patients (88.3% and 61.0% versus 71.8% and 42.3%, respectively). The weight loss percentages reported by our lockdown patients are similar to the mean 8% weight loss reported in a similar time frame by a recent review of lifestyle modification approaches for the treatment of obesity in adults [31], although those reported by our control patients were higher. These data seem to indicate that intensive residential CBT-OB, designed to facilitate initial adherence to dietary restriction in a protected environment and address some specific cognitive–behavioral obstacles to weight loss, obtains similar outcomes to standard lifestyle modification of obesity if administered in adverse conditions, but a better outcome when conducted under normal circumstances.

The third finding from this study is that both groups showed a significant reduction in binge-eating episodes, with more than 80% of patients reporting no binge-eating episodes in the last 28 days at 6 months from discharge. Although several studies have indicated a worsening of eating behaviors and habits in patients with obesity during the COVID-19 lockdown [32,33], our study did not confirm that binge-eating episodes were more frequent in lockdown versus control patients treated with intensive residential CBT-OB.

Our fourth finding concerns patients’ responses to our ad hoc COVID-19 interview. In general, patients who experienced lockdown reported that both their weight management and quality of life worsened during that time. However, about 50% of the 101 respondents declared that they continued to practice the regular eating procedure, stick to their daily calorie goals, and maintain an active lifestyle. These data only partially support previous findings that lockdown had a significant negative impact on patients with obesity in terms of their eating habits [33]. Indeed, albeit self-reported, our data indicate that half of our patients continued to practice healthy weight management behaviors during lockdown.

Nevertheless, our study did suffer from certain limitations. First, the self-reported bodyweight at 6-month follow-up could have overestimated the amount of weight lost but was a factor common to both groups. Second, the retrospective nature of the COVID-19 interview may have been influenced by recall bias. Third, a longer period of follow-up would have been desirable for determining the weight change in the longer term. Fourth, the very particular setting, with an initial intensive residential period—rarely used in obesity treatment in the community—makes it difficult to extend the results to the normal outpatient setting. Finally, the relatively small sample size and the absence of specific variables associated with the COVID-19 lockdown do not permit evaluating the mechanisms that hindered the weight management of patients with obesity exposed to the COVID-19 lockdown.

That being said, the study had several strengths. First, to our knowledge, this is the first study comparing patients with obesity exposed to lockdown with gender-, age-, and BMI-matched patients who received the same treatment. This design is the best source of reliable data on the impact of COVID-19 lockdown on weight management in patients treated for obesity. Second, the treatment was a well-validated intervention delivered in a real-world setting and uses some original procedures to personalize the intervention and to address the specific cognitive processes that our previous research has found to be associated with attrition, weight loss, and weight maintenance [34]. Third, the fact that the COVID-19 and 6-month follow-up interviews were conducted by expert clinicians not involved in the treatment, rather than relying on self-report questionnaires, enabled us to screen the responses (subjectively) for unreliable data and better investigate and understand the patients’ perspectives.

## 5. Conclusions

Our data suggest that patients with severe obesity exposed to COVID-19 lockdown lost less weight than those not exposed to lockdown treated with the same intensive residential CBT-OB. Control patients also perceived a better mood status, but a comparable general health status to lockdown patients. Despite these differences, lockdown patients had achieved a significant and healthy mean weight loss at 6 months, similar to that yielded by standard lifestyle modification for obesity in non-pandemic periods. Moreover, they reported a significant reduction in binge-eating episodes, and about half declared continuing to adhere to CBT-OB weight management strategies and procedures. This suggests that even during adverse events such as the COVID-19 pandemic, a specific treatment for obesity, namely intensive residential CBT-OB, seems to be able to help patients to maintain successful weight management. Variables associated with COVID-19 lockdown to evaluate in future studies should include anxiety about infection, loss of structure and routine, social isolation, financial stress, childcare pressure, feeling less deserving of therapeutic help, limited availability of “healthy” foods, bulk buying and hoarding of foods, and restriction of exercise. Finally, future studies with a robust study design should evaluate the longer-term impact of COVID-19 lockdown in patients with obesity.

## Figures and Tables

**Table 1:** Demographic and clinical characteristics of 129 patients with severe obesity exposed to COVID-19 lockdown and 129 gender-, age-, and body mass index-matched patients not exposed to COVID-19 lockdown (controls). Data are presented as mean (SD) or as number (%).

	Lockdown Patients (*n* = 129)	Control Patients (*n* = 129)	*t* Test, Mann–Whitney or Chi-Squared Test	*p*-Value
Gender, % women	90 (69.8%)	90 (69.8%)	--	--
Age, y	57.0 (14.2)	56.5 (14.0)	Z = 0.33	0.742
Body Mass Index, kg/m^2^	41.6 (8.3)	42.2 (8.1)	Z = 0.72	0.474
EDE-Q global score	2.5 (1.2)	2.6 (1.1)	t = 0.92	0.354
EDE-Q restraint	1.7 (1.5)	1.6 (1.3)	Z = 0.40	0.689
EDE-Q eating concern	1.7 (1.4)	1.8 (1.3)	Z = 0.77	0.443
EDE-Q weight concern	3.0 (1.4)	3.2 (1.3)	Z = 1.36	0.174
EDE-Q shape concern	3.5 (1.6)	3.9 (1.6)	Z = 1.85	0.065
EDE-Q objective binge-eating episodes, mean (SD), if present	8.2 (8.0)	11.5 (19.5)	χ^2^ = 1.45	0.148
EDE-Q self-induced vomiting, mean (SD), if present	5.6 (3.8)	5.8 (4.6)	χ^2^ = 0.07	0.942
EDE-Q laxative misuse, mean (SD), if present	4.0 (2.6)	14.0 (10.9)	χ^2^ = 1.52	0.166
EDE-Q excessive exercise, mean (SD), if present	8.2 (6.2)	6.0 (5.6)	χ^2^ = 1.23	0.227
SCL-90-R global score	0.83 (0.54)	0.78 (0.63)	Z = 1.18	0.237

EDE-Q = Eating Disorder Examination Questionnaire; SCL-90-R= Symptom Check List-90-Revised.

**Table 2:** Body mass index, weight change, and 5 and 10% weight change from baseline to 6-month follow-up in patients with obesity exposed and not exposed (controls) to COVID-19 lockdown. Data from completer and intention-to-treat analysis. For repeated measure ANOVA: Mauchly’s sphericity tests were >0.05. Sphericity was assumed.

Completer Analysis								
	Admission		Discharge		6-Month Follow-Up			
	Lockdown Patients (*n* = 71)	Control Patients (*n* = 77)	Lockdown Patients (*n* = 71)	Control Patients (*n* = 77)	Lockdown Patients (*n* = 71)	Control Patients (*n* = 77)	Repeated-Measures ANOVA	
							Time	TimeXGroup
Bodyweight in kg, mean (SD)	111.7 (22.9)	117.0 (22.2)	106.5 (21.7)	111.8 (21.1)	101.1 (22.0)	101.6 (19.7)	<0.001	0.002
Body mass index in kg/m^2^, mean (SD)	40.9 (7.4)	43.2 (6.8)	39.1 (7.1)	41.3 (6.6)	37.0 (7.2)	37.6 (6.6)	<0.001	0.002
							*t*-test; *p*-value discharge	*t*-test; *p*-value 6-month follow-up
Weight loss from admission in kg, mean (SD)	--	--	5.2 (3.5)	5.2 (2.8)	10.6 (9.2)	15.3 (9.3)	0.16; 0.872	3.09; 0.002
Percentage weight loss from admission, mean (SD)	--	--	4.6 (3.1)	4.3 (2.0)	9.4 (7.5)	13.0 (7.1)	0.65; 0.514	2.94; 0.004
							Chi-squared test; *p*-value discharge	Chi-squared test; *p*-value 6-month follow-up
≥5% weight loss from admission, *n* (%)	--	--	20 (28.2%)	25 (32.5%)	51 (71.8%)	68 (88.3%)	0.32; 0.570	6.37; 0.012
≥10% weight loss from admission, *n* (%)	--	--	1 (1.4%)	1 (1.3%)	30 (42.3%)	47 (61.0%)	0.003; 0.954	5.22; 0.022
**Intention-to-Treat Analysis**								
	**Admission**		**Discharge**		**6-Month Follow-Up**		**Repeated-Measures ANOVA**	
							**Time**	**TimeXGroup**
	**Lockdown Patients (*n* = 129)**	**Control Patients (*n* = 129)**	**Lockdown Patients (*n* = 129)**	**Control Patients (*n* = 129)**	**Lockdown Patients (*n* = 129)**	**Control Patients (*n* = 129)**		
Bodyweight in kg, mean (SD)	112.5 (23.0)	115.5 (24.7)	107.4 (21.8)	110.5 (23.4)	101.3 (22.6)	101.2 (22.4)	<0.001	<0.001
Body mass index in kg/m^2^, mean (SD)	41.6 (8.2)	42.2 (8.1)	39.9 (7.9)	40.4 (7.7)	37.5 (8.3)	37.1 (7.8)	<0.001	<0.001
							*t*-test; *p*-value discharge	*t*-test; *p*-value 6-month follow-up
Weight loss from admission in kg, mean (SD)	--	--	5.06 (2.9)	5.07 (2.8)	11.1 (10.0)	14.4 (10.2)	0.12; 0.908	8.45; <0.001
Percentage weight loss from admission, mean (SD)	--	--	4.5 (2.4)	4.3 (2.1)	9.9 (8.6)	12.3 (8.2)	1.88; 0.060	7.36; <0.001
							Chi-squared test; *p*-value discharge	Chi-squared test; *p*-value 6-month follow-up
≥5% weight loss from admission, *n* (%)	--	--	37 (28.7%)	42 (32.6%)	92 (71.3%)	106 (82.2%)	5.02; 0.025	44.6; <0.001
≥10% weight loss from admission, *n* (%)	--	--	1 (0.8%)	1 (0.8%)	59 (45.8%)	76 (58.9%)	--	48.6; <0.001

**Table 3:** Binge-eating episodes at admission and at 6-month follow-up in patients with severe obesity exposed and not (controls) to COVID-19 lockdown. Data are presented for completers.

	**Admission**		**6-Month Follow-Up**			
	**Lockdown Patients (*n* = 71)**	**Control Patients (*n* = 77)**	**Lockdown Patients (*n* = 71)**	**Control Patients (*n* = 77)**	**Chi-Squared Test; *p*-Value between Groups**	**Sign Test; *p*-Value within Groups**
Objective binge-eating episodes, *n* (%) No episodes Fewer than 1 episode per week 1 episode per week 2–3 episodes per week 4–7 episodes per week 8–13 episodes per week 14 or more episodes per week	24 (33.8) 16 (22.5) 17 (23.9) 8 (11.3) 6 (8.5) 0 0	23 (30.3) 15 (19.7) 16 (21.1) 10 (13.2) 11 (14.5) 0 1 (1.3)	58 (81.7) 6 (8.5) 3 (4.2) 2 (2.8) 1 (1.4) 1 (1.4) 0	66 (86.8) 6 (7.9) 2 (2.6) 2 (2.6) 0 0 0	Admission: 2.61; 0.760 6-month follow-up: 2.55; 0.769	Lockdown: 5.18; <0.001 Control: 6.72; <0.001

**Table 4:** COVID-19 phone interview scores in 101 lockdown patients.

During the COVID-19 Lockdown…	
…were you shielding	No, *n* = 99/101 (98%) Yes, *n* = 2/101 (2%)
…did you stop working or lose your job	I was not working before the lockdown, *n* = 45/101 (44.5%) No, *n* = 35/56 (62.5%) Yes, *n* = 19/56 (30.4%)
…did you continue to go to work outside your home	No, *n* = 17/35 (48.6%) Yes, *n* = 17/35 (48.6%) Both, *n* = 1/35 (2.8%)
…did you continue to work remotely from home (smart working)	No, *n* = 17/35 (48.6%) Yes, *n* = 17/35 (48.6%) Both, *n* = 1/35 (2.8%)
…did you worry about not having enough food available	No, *n* = 94/101 (93.1%) Yes, *n* = 7/101 (6.9%)
…did you accumulate more food than you normally would	No, *n* = 67/101 (66.3%) Yes, *n* = 34/35 (33.7%)
… how often did you follow the “regular eating” procedure (3 planned meals + 2 snacks and not eating between)	Never, *n* = 22/101 (21.8%) Rarely, *n* = 8/101 (7.9%) Sometimes, *n* = 21/101 (20.8%) Often, *n* = 22/101 (21.8%) Always, *n* = 28/101 (27.7%)
…how often did you stick to your calorie goals set at Villa Garda	Never, *n* = 26/101 (25.7%) Rarely, *n* = 9/101 (8.9%) Sometimes, *n* = 17/101 (16.8%) Often, *n* = 18/101 (17.8%) Always, *n* = 31/101 (30.7%)
… how often did you exercise	Never, *n* = 29/101 (28.7%) Rarely, *n* = 10/101 (9.9%) Sometimes, *n* = 13/101 (12.9%) Often, *n* = 26/101 (25.7%) Always, *n* = 23/101 (22.8%)

## Data Availability

The data presented in this study are available on request from the corresponding author.

## Appendix A

COVID-19 Interview:

I am Dr. … of Villa Garda Hospital. You have been treated in our Unit.

Could I have your permission to ask you some questions over the phone to assess the effects of the COVID-19 emergency on your health?

Can we start with the questions or do you need further clarification?

001 Patient Number (ID)
003 Interview Date
004 Interviewer’s Name
005 Interview Outcome (1 = tracked down; 2 = refuses telephone contact; 3 = not found/unreliable data)
006 Have you ever been diagnosed with COVID-19? (0 no, 1 yes)
  If yes, end the call by thanking
  If no, continue the call
007 During the COVID-19 lockdown, were you shielding?
008 During the COVID-19 lockdown, did you stop working or lose your job? (0 no, 1 yes, I was not working before the lockdown)
  - During the COVID-19 lockdown, did you continue to go to work outside your home? (0 no, 1 yes)
  - During the COVID-19 lockdown, did you continue to work remotely from home (smart working)? (0 no, 1 yes)
009 During the COVID-19 lockdown, did you worry about not having enough food available? (0 no, 1 yes)
0010 During the COVID-19 lockdown, did you accumulate more food than you normally would? (0 no, 1 yes)
0011 During the COVID-19 lockdown, how often did you follow the “regular eating” procedure (3 planned meals + 2 snacks and not eating between)? (0 = never; 1 = rarely; 2 = sometimes; 3 = often; 4 = always)
0012 During the COVID-19 lockdown, how often did you stick to your calorie goals set at Villa Garda? (0 = never; 1= rarely; 2= sometimes; 3= often; 4= always)
0013 During the COVID-19 lockdown, how often did you exercise? (0= never; 1= rarely; 2= sometimes; 3= often; 4= always)
